# Well-Defined Palladium Nanoparticles Supported on Siliceous Mesocellular Foam as Heterogeneous Catalysts for the Oxidation of Water

**DOI:** 10.1002/chem.201406279

**Published:** 2015-03-16

**Authors:** Oscar Verho, Torbjörn Åkermark, Eric V Johnston, Karl P J Gustafson, Cheuk-W Tai, Henrik Svengren, Markus D Kärkäs, Jan-E Bäckvall, Björn Åkermark

**Affiliations:** [a] Department of Organic Chemistry, Arrhenius Laboratory, Stockholm University106 91 Stockholm (Sweden); [b] Department of Materials and Environmental Chemistry, Arrhenius Laboratory, Stockholm University106 91, Stockholm (Sweden); [c] Berzelii Center EXSELENT on Porous Materials, Arrhenius Laboratory, Stockholm University106 91, Stockholm (Sweden)

**Keywords:** heterogeneous catalysis, mesoporous materials, nanoparticles, palladium, water splitting

## Abstract

Herein, we describe the use of Pd nanoparticles immobilized on an amino-functionalized siliceous mesocellular foam for the catalytic oxidation of H_2_O. The Pd nanocatalyst proved to be capable of mediating the four-electron oxidation of H_2_O to O_2_, both chemically and photochemically. The Pd nanocatalyst is easy to prepare and shows high chemical stability, low leaching, and recyclability. Together with its promising catalytic activity, these features make the Pd nanocatalyst of potential interest for future sustainable solar-fuel production.

## Introduction

In attempts to develop a system for the conversion of solar energy into fuel, significant work has been devoted to the preparation of efficient catalysts for the oxidation of H_2_O because this reaction constitutes the main impediment in the construction of an economical and practically viable photosynthetic fuel cell.[1] Extensive research in this area has resulted in the development of various molecular (homogeneous) water-oxidation catalysts (WOCs) comprised of Ru[2–10] and Ir[11–13] species or of the more abundant first-row transition metal species Fe[14–16] Mn,[17–20] Co,[21,22] and Cu.[23,24] However, these catalysts are all associated with oxidative degradation and/or deactivation,[25,26] which significantly decreases their longevity and applicability in photosynthetic devices. Consequently, recent research has been dedicated to heterogeneous catalysts because these catalysts often exhibit high stability and recyclability and are easy to integrate into solar-fuel devices.[27,28] These efforts have generated various inorganic polyoxometalates (POMs)[29–31] and a variety of different transition metal oxides based primarily on Ru,[32–34] Ir,[35,36] Mn,[37–41] and Co,[42–48] which have been shown to be efficient and stable WOCs. Of these catalysts, those based on small nanoparticle species hold greater potential because their higher surface-to-volume ratios result in improved catalytic efficiency.

So far, the utilization of nanostructured materials has yielded several catalytic systems for H_2_O-oxidation activity, which display higher efficiencies relative to their unsupported or bulk metal oxide material counterparts.[49] However, there is still room for improvement because the majority of these heterogeneous materials produce O_2_ with a turnover number (TON) lower than 1 (TON is based on the moles of produced O_2_/total moles of metal atoms in the catalyst). In view of the seminal results obtained with heterogeneous materials involving metal oxide catalysts, the examination of transition metal nanoparticle-based WOCs constitutes a promising research frontier within H_2_O-oxidation catalysis.

In nanoparticle-based WOCs, the nanoparticles are usually immobilized on a support. The type of support to which these nanoparticles are immobilized has been demonstrated to have a critical impact on the catalyst performance by offering stabilization of the nanoparticles and preventing them from agglomerating. Among the numerous materials available, mesoporous silica materials are some of the most well-studied and frequently used supports for the immobilization of metal nanoparticles. The main advantages of mesoporous silica supports are the straightforward synthesis, low cost, chemical stability, adjustable pore structure, and the possibility to graft them with a wide range of functional groups.[32,42,44]

Palladium catalysts have found extensive use in organic synthesis, but their use in H_2_O-oxidation catalysis is rare. Thampi and Grätzel showed in 1990 that palladium oxide immobilized on various metal oxide supports could function as a competent heterogeneous WOC when driven by the strong oxidant Ce^IV^.[50] More recently, Kwon et al. reported on the anchoring of sub-nanometer palladium oxide clusters onto an ultra-nanocrystalline electrode and the application of these assemblies for electrochemically driven H_2_O oxidation.[51] In accordance with previous research within the field of nanocatalysis,[52] we envisioned that it would also be possible to achieve a higher H_2_O-oxidation activity by the use of small and well-defined Pd nanoparticles. For this purpose, we studied a nanocatalyst that consists of Pd nanoparticles immobilized on an amino-functionalized mesocellular foam (AmP-MCF), which had previously been employed by our group for a wide range of organic transformations.[53–56] This catalyst constitutes a particularly suitable model system for studying the H_2_O-oxidation activity of Pd nanoparticles because the mesoporosity of the support allows for a high dispersion of very small Pd nanoparticles in a narrow size range. To our delight, this catalyst proved to be capable of mediating the four-electron oxidation of H_2_O to O_2_, both chemically and photochemically. Moreover, the developed Pd nanocatalyst, with its tunability, potential ease of interfacing with electrode systems, and oxidative stability, offers additional advantages over current homogeneous catalysts and provides a possible way of realizing fast and robust WOCs for sustainable solar-fuel production.

## Results and Discussion

### Catalyst design and characterization

**Figure 1:**
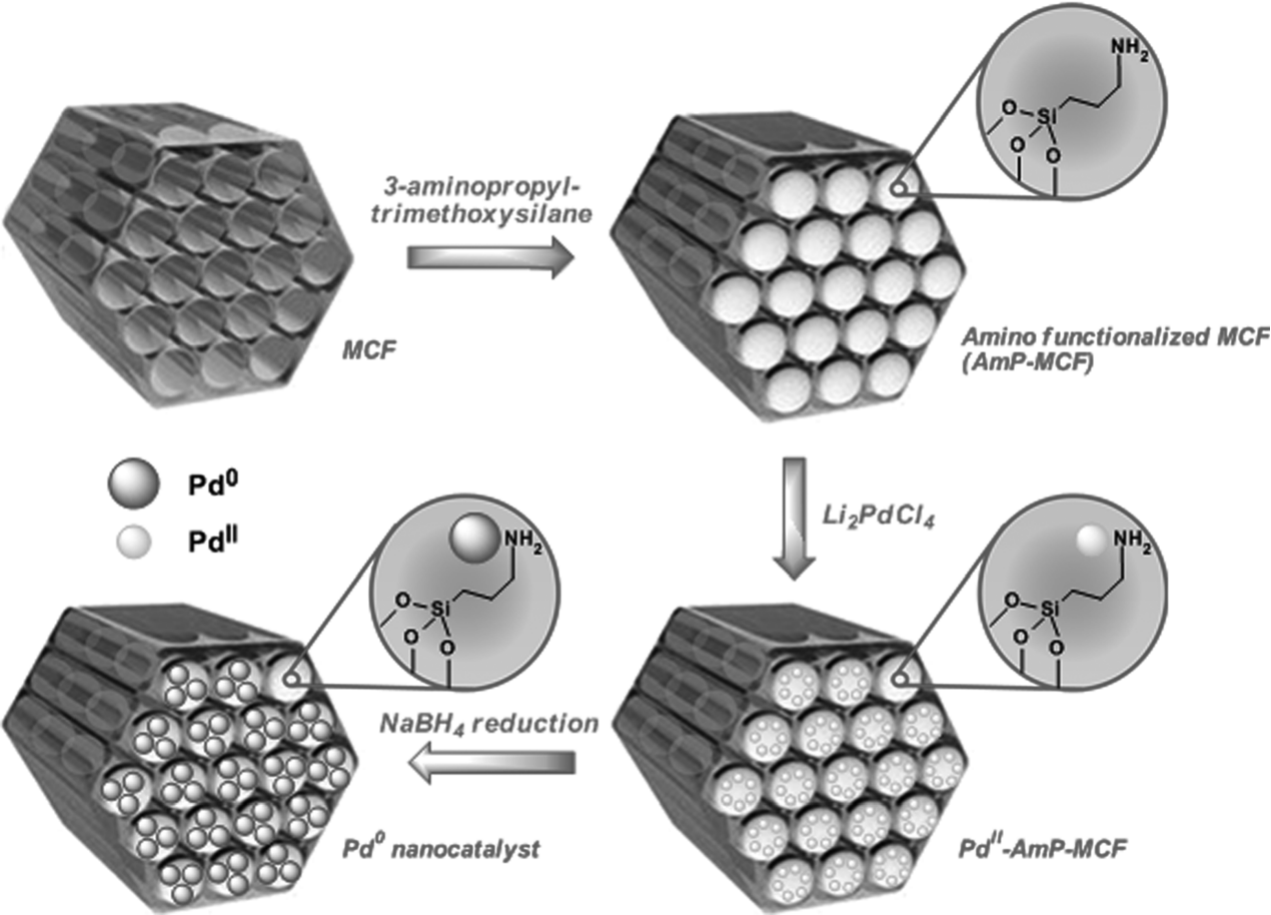
Synthetic route to the Pd nanocatalyst.

The synthesis of the Pd nanocatalyst was performed according to a previously reported protocol, which is outlined in Figure 1 and described in detail in the Supporting Information.[55,56] Briefly, the MCF material is grafted with aminopropyl groups in the first step by treatment with 3-aminopropyltrimethoxysilane in toluene at reflux. The amino-functionalized MCF is then impregnated with Li_2_PdCl_4_ in H_2_O (pH 9) at room temperature to give the Pd^II^-AmP-MCF precatalyst. Finally, the heterogeneously supported Pd^II^ species is reduced by ten equivalents of NaBH_4_, thus giving the desired Pd nanocatalyst.

The morphology, size, and structure of pristine AmP-MCF and the Pd nanocatalyst were characterized by a toolbox of analytical techniques, including N_2_ adsorption/desorption isotherms, IR spectroscopy, high-angle annular dark-field scanning transmission electron microscopy (HAADF-STEM), X-ray photoelectron spectroscopy (XPS), elemental analysis, high-resolution TEM, and convergent-beam electron diffraction (CBED). By employing the Barrett–Joyner–Halenda method of isotherm analysis, the average pore size and window size of the pristine AmP-MCF were determined to be 26.0 and 13.6 nm, respectively.[55] The specific pore volume and surface area of the pristine AmP-MCF were calculated to 1.65 cm^3^ g^−1^ and 380 m^2^ g^−1^, respectively, by using the Brunauer–Emmett–Teller (BET) surface-area analysis.[55] The presence of the grafted aminopropyl groups on both the pristine AmP-MCF and the Pd nanocatalyst was confirmed by IR spectroscopic analysis, which showed the appearance of characteristic peaks in the regions 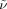
=2924–2926 (C_sp3_—H stretch), 2853–2854 (C_sp3_—H stretch), and 1377–1378 cm^−1^ (C_sp3_—N bend; see Figures S1–S3 in the Supporting Information).[55]

**Figure 2:**
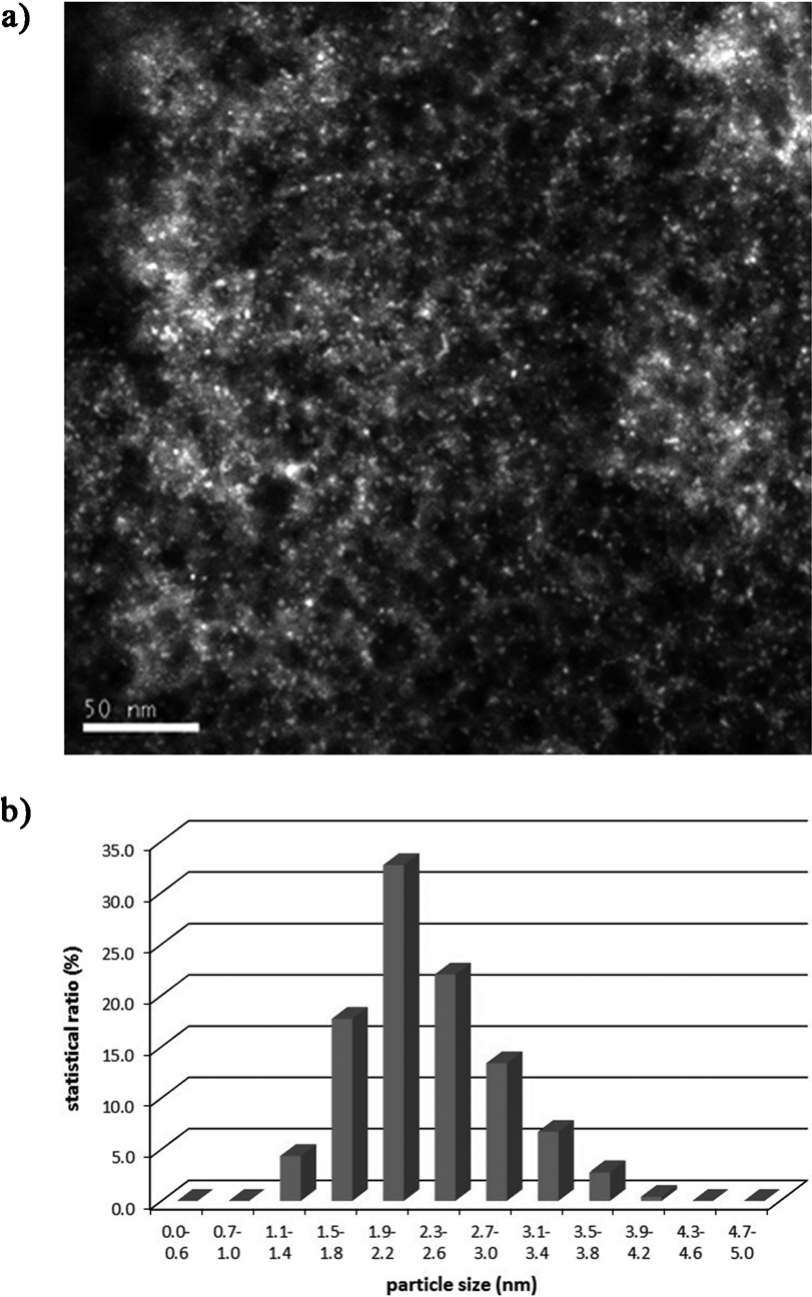
Structure and particle-size distribution of the Pd nanocatalyst. a) Representative image of the Pd nanocatalyst taken by means of HAADF-STEM. b) Particle-size distribution for the Pd nanocatalyst.

Elemental analysis by means of inductively coupled plasma-optical emission spectroscopy (ICP-OES) allowed for the quantification of the N content of the pristine AmP-MCF, which was 1.9 wt %. The Pd nanocatalyst was also analyzed by means of ICP-OES, which determined the Pd and N contents to be 7.9 and 1.4 wt %, respectively. Isotherm analysis of the Pd nanocatalyst revealed average pore and window sizes of 18.4 and 12.0 nm, respectively, whereas BET surface-area analysis showed a specific pore volume and surface area of 1.55 cm^3^ g^−1^ and 341 m^2^ g^−1^, respectively. The Pd nanocatalyst was further characterized by HAADF-STEM to allow for an assessment of the size and distribution of the Pd nanoparticles, thus revealing that the catalyst possesses a highly dispersed pattern of nanoparticles in a narrow size range. A statistical determination of the nanoparticle-size distribution by means of HAADF-STEM showed that the majority of the nanoparticles are within the size range 1.5–2.6 nm (Figure 2). In our previous study,[55] we showed by means of XPS that the nanocatalyst consisted predominantly of Pd^0^ centers, with only minor amounts of Pd^II^ centers. In addition, the combined use of CBED and high-resolution TEM showed that the Pd particles are nanocrystalline, thus suggesting that the atoms within the nanoparticles were structured in a uniform and ordered manner.

### Catalyst evaluation by using chemical oxidants

**Figure 3:**
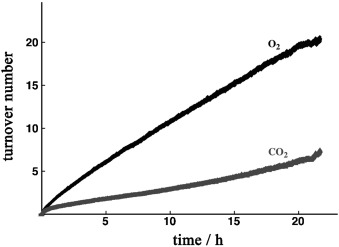
Kinetics of the O_2_ evolution by using Ce^IV^(NH_4_)_2_(NO_3_) (CAN) as the chemical oxidant. Kinetic curves for the chemical O_2_ and CO_2_ evolution versus time by the Pd nanocatalyst with CAN as the chemical oxidant. Conditions: the Pd nanocatalyst (0.60 mg, 7.9 wt % Pd, 0.45 μmol Pd) and CAN (61 mg, 0.11 mmol) were placed and mixed in the reaction chamber, H_2_O (1 mL) was added to this mixture, and the O_2_ measurements were immediately initiated.

The Pd nanocatalyst was initially assessed as a catalyst for chemical H_2_O oxidation by using the strong one-electron oxidant ceric ammonium nitrate (CAN, Ce^IV^). In this study, the evolved gaseous products were measured by using real-time mass-spectrometric analysis, a technique that we have used previously for the evaluation of molecular WOCs.[3] Upon the addition of H_2_O to a solid mixture of the Ce^IV^ oxidant and the Pd nanocatalyst, an immediate evolution of O_2_ was triggered, which was sustained for 20 hours and reached a TON of approximately 20 based on the total amount of Pd (Figure 3). We also observed significant evolution of CO_2_ during these initial catalytic experiments, which most likely originated from oxidative degradation of the AmP-ligand backbone. However, it is not possible to exclude that decomposition of solvent residues and other organic material that were still trapped within the cavities of the MCF support or remained adsorbed onto the Pd surface from the catalyst synthesis accounts for some of the detected CO_2_. The O_2_-evolution activity of the Pd nanocatalyst during the first 20 hours seemed to be unaffected by the CO_2_ evolution (Figure 3). This initial catalytic experiment with the Ce^IV^ oxidant demonstrates that the Pd nanocatalyst is indeed active and stable under the catalytic H_2_O-oxidation conditions, which motivated us to continue the catalytic evaluation.

Unfortunately, the Ce^IV^ oxidant is not viable for practical use in future devices for solar-to-chemical energy conversion because the Ce^IV^ ion has a low absorption in the visible region and cannot be photochemically regenerated from the Ce^III^ ion. A feasible approach to enable light-driven processes would be to drive H_2_O oxidation with [Ru(bpy)_3_]^3+^-type (byp=2,2′-bipyridine) oxidants [Eq. (1)], which can be photogenerated from the corresponding [Ru(bpy)_3_]^2+^-type photosensitizer complexes. To realize this scenario, the redox potential of the WOC must be lower than that of the photosensitizer to make it thermodynamically favorable to carry out the four-electron oxidation of H_2_O. This situation is not easily accomplished and the majority of the reported homogeneous WOCs require Ce^IV^ to drive the oxidation of H_2_O. Therefore, it was of interest to establish if the developed Pd nanocatalyst could mediate the catalytic H_2_O oxidation by the use of the one-electron oxidant [Ru(bpy)_3_]^3+^ (*E*_1/2_ (Ru^III^/Ru^II^)=1.26 V vs. the normal hydrogen electrode (NHE)).

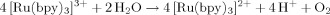
(1)

**Figure 4:**
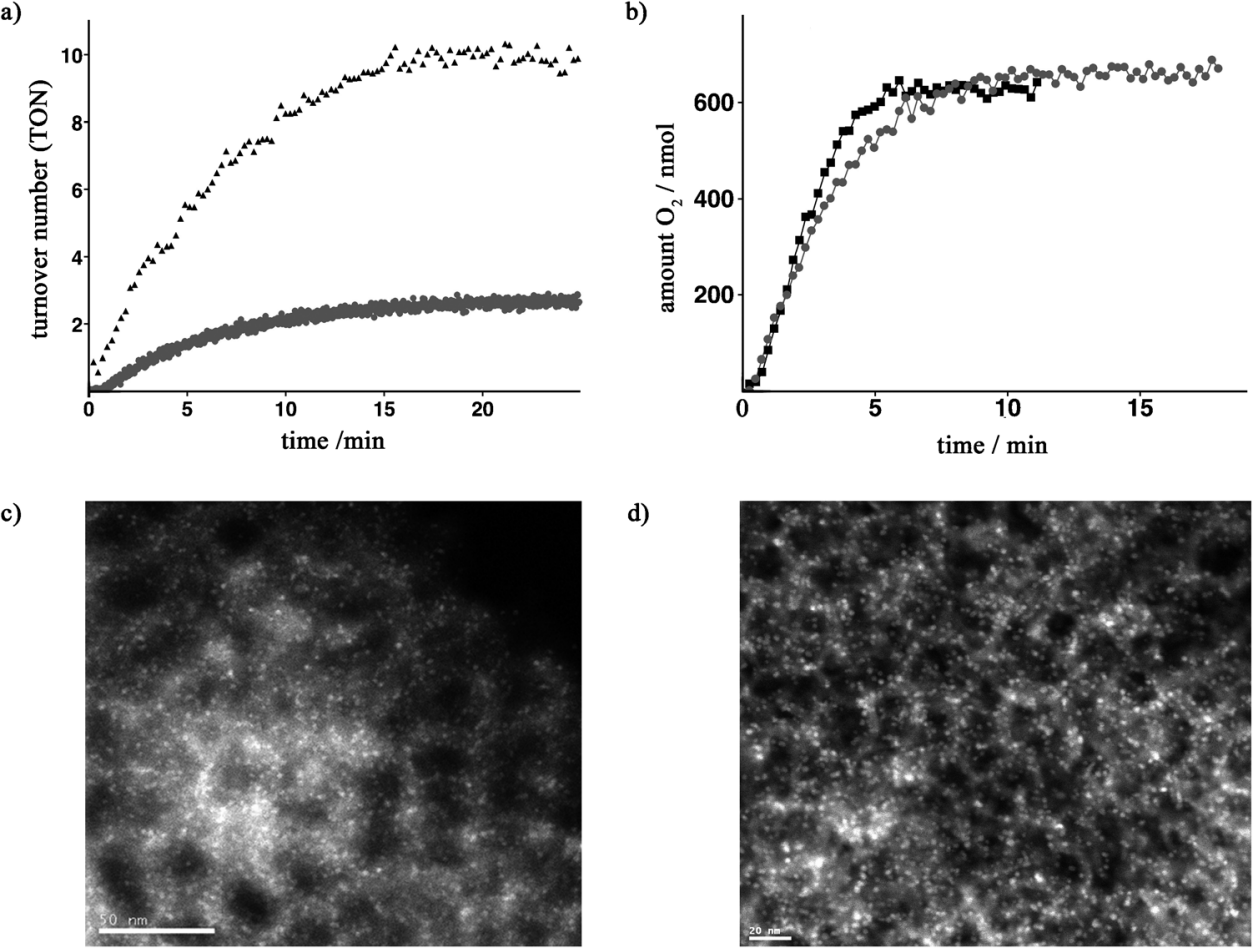
Kinetics of the O_2_ evolution by using [Ru(bpy)_3_](PF_6_)_3_ as the chemical oxidant and HAADF-STEM images of the Pd nanocatalyst. a) Kinetic curves for the O_2_ evolution by the Pd nanocatalyst versus time. Conditions: an aqueous phosphate buffer solution (0.1 M, pH 7.2, 0.5 mL) was added to the oxidant [Ru(bpy)_3_](PF_6_)_3_ and the Pd nanocatalyst. (▴) Pd nanocatalyst (20 μg Pd nanocatalyst, 7.9 wt % Pd, 15.1 nmol Pd) and [Ru(bpy)_3_](PF_6_)_3_ (7.0 mg, 7.0 μmol); (•) Pd nanocatalyst (0.25 mg Pd nanocatalyst, 7.9 wt % Pd, 0.19 μmol Pd) and [Ru(bpy)_3_](PF_6_)_3_ (45 mg, 45 μmol). b) Kinetic curves for the O_2_ evolution by using the recycled Pd nanocatalyst versus time. Conditions: an aqueous phosphate buffer solution (0.1 M, pH 7.2, 0.50 mL) was added to the oxidant [Ru(bpy)_3_](PF_6_)_3_ (5.3 mg, 5.3 μmol) and the Pd nanocatalyst (0.36 mg Pd nanocatalyst, 7.9 wt % Pd, 0.27 μmol Pd). (▪) First run, (•) second run. c) HAADF-STEM image of the Pd nanocatalyst (scale bar=50 nm). d) HAADF-STEM image of the recovered Pd nanocatalyst after H_2_O-oxidation catalysis (scale bar=20 nm).

The catalytic experiments were carried out in buffered aqueous solutions under neutral conditions (0.1 M phosphate buffer, pH 7.2). No O_2_ evolution was observed in the absence of the Pd nanocatalyst or with pristine AmP-MCF. Instead, the [Ru(bpy)_3_]^3+^ oxidant was spontaneously reduced and partially degraded within less than an hour. However, catalytic H_2_O oxidation with [Ru(bpy)_3_]^3+^ was achieved in the presence of the Pd nanocatalyst with the concomitant generation of O_2_ with high TON and turnover frequency (TOF) values of up to 10 and 2.7×10^−2^ s^−1^, respectively (Figure 4 a).[57] The yield of O_2_ based on the [Ru(bpy)_3_]^3+^ oxidant is given in Table S1 (see the Supporting Information). The low O_2_/[Ru(bpy)_3_]^3+^ yields at neutral pH are due to a competing decomposition of the [Ru(bpy)_3_]^3+^ oxidant, which is a process that does not produce any O_2_ and could explain the rapid termination of O_2_ evolution that occurred after 15 minutes for the studied system.

To confirm that H_2_O is the sole source of the evolved O_2_, catalytic experiments were conducted with isotopically labeled H_2_^18^O (16 %). The proportionally enriched O_2_ was measured by means of real-time mass-spectrometric analysis, and the ratio of the isotopologues ^18, 18^O_2_/^16, 16^O_2_ and ^18, 16^O_2_/^16, 16^O_2_ confirmed that the oxygen atoms in the generated O_2_ were derived only from H_2_O (see Figure S5 in the Supporting Information). To verify the stability of the Pd nanocatalyst in the H_2_O-oxidation experiments with [Ru(bpy)_3_]^3+^, the catalyst was recycled after a catalytic H_2_O-oxidation reaction and reused under identical conditions. The recovered Pd nanocatalyst displayed comparable activity to that of the unused catalyst, thus confirming its robustness under the catalytic conditions (Figure 4 b). Further support for the stability of the Pd nanocatalyst was provided by HAADF-STEM analysis, which showed that the recovered catalyst exhibited a similar nanostructure to that of the unused catalyst, without any observable change in either the size or dispersion pattern of the nanoparticles (Figure 4 c, d). To establish the extent of Pd leaching, liquid aliquots were withdrawn from the first and second cycle of the recycling study for ICP-OES analysis (3.76 and 0.94 nmol, respectively). These values correspond to 1.4 and 0.4 % of the total amount of Pd that was used in each experiment, thus further demonstrating the stability of the Pd nanocatalyst under the employed catalytic conditions.

### Photocatalytic H_2_O oxidation

**Figure 5:**
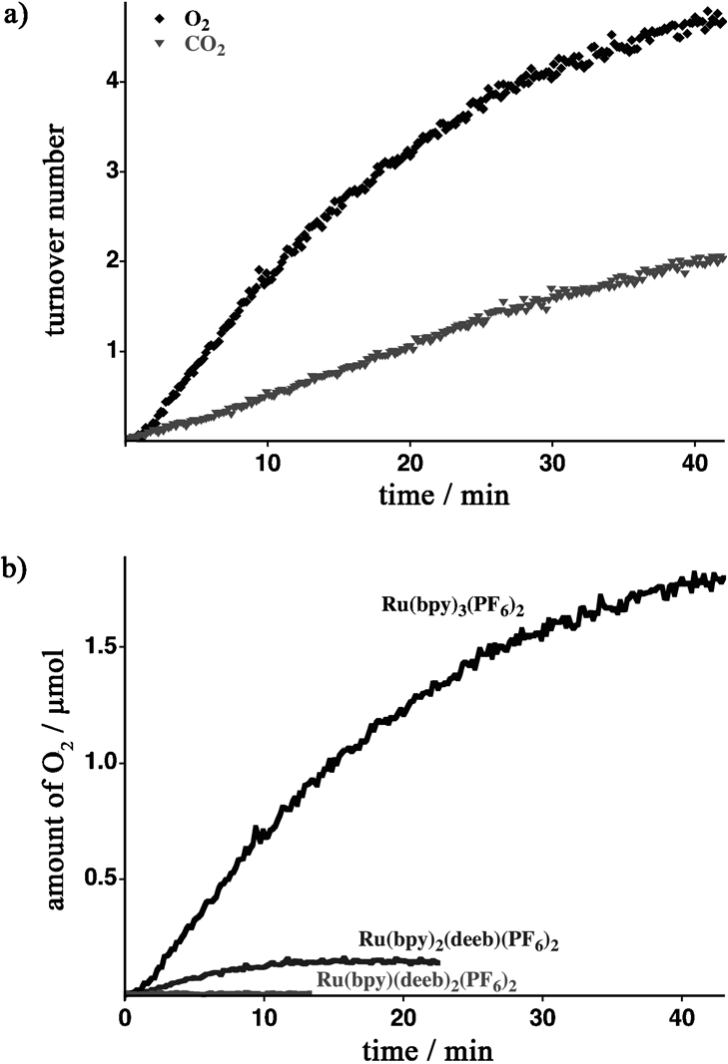
Kinetic curves for photochemical O_2_ evolution by the Pd nanocatalyst versus time. a) Photochemical H_2_O oxidation by using [Ru(bpy)_3_](PF_6_)_2_ as a photosensitizer. Conditions: the experiments were performed in an aqueous phosphate buffer solution (0.1 M, pH 7.2) containing [Ru(bpy)_3_](PF_6_)_2_ (400 μM, 0.20 μmol) as a photosensitizer, sodium persulfate (24.4 mM, 12.2 μmol) as the sacrificial electron acceptor, and the Pd nanocatalyst (0.50 mg, 7.9 wt % Pd, 0.37 μmol Pd). b) Photochemical H_2_O oxidation with different photosensitizers. Conditions: the experiments were carried out in an aqueous phosphate buffer solution containing the photosensitizer (400 μM, 0.20 μmol), sodium persulfate (24.4 mM, 12.2 μmol), and the Pd nanocatalyst (0.50 mg, 7.9 wt % Pd, 0.37 μmol Pd).

We also investigated the catalytic system photochemically by using [Ru(bpy)_3_]^3+^, which was continuously photochemically generated from the corresponding [Ru(bpy)_3_]^2+^ photosensitizer. The photochemical experiments were conducted by using a three-component system, which consisted of the Pd nanocatalyst, [Ru(bpy)_3_]^2+^ as the photosensitizer, and sodium persulfate as the sacrificial electron acceptor. The evolution of O_2_ (and minor amounts of CO_2_) occurred upon illumination with visible light and was monitored by means of real-time mass spectrometry, which showed a TON of approximately 5 (based on the total amount of Pd) over 40 minutes (Figure 5 a). This TON corresponds to an overall efficiency of greater than 30 % with respect to the persulfate ion.

The results obtained from the photochemical study shows that the Pd nanocatalyst is relevant for incorporation into artificial photosynthetic devices for solar-fuel production by using previously reported strategies that involve, for instance, the co-immobilization of a WOC unit and a chromophore on a conducting material.[58–60]

In attempts to further improve the efficiency of the present photochemical protocol and provide insight in the catalytic mechanism, the two photosensitizers [Ru(bpy)_2_(deeb)]^2+^ (1.40 V vs. NHE; deeb=diethyl-2,2′-bipyridine-4,4′-dicarboxylate) and [Ru(bpy)(deeb)_2_]^2+^ (1.54 V vs. NHE) with higher redox potentials were tested and compared to the reaction involving [Ru(bpy)_3_]^2+^ (Figure 5 b). Based on previous reports,[61,62] the formation of high-valent Pd species, which are proposed to participate in the catalytic cycle, should increase with an increased redox potential of these photosensitizers. In constrast to the [Ru(bpy)_2_(deeb)]^2+^ and [Ru(bpy)(deeb)_2_]^2+^ photosensitizers, the weaker [Ru(bpy)_3_]^2+^ photosensitizer would not be expected to have the required redox potential to bring the Pd nanocatalyst to the Pd^IV^ state. Interestingly, the weakest photosenzitizer [Ru(bpy)_3_]^2+^ afforded large amounts of O_2_, whereas the two stronger photosensitizers [Ru(bpy)_2_(deeb)]^2+^ and [Ru(bpy)(deeb)_2_]^2+^ gave rise to no or only trace amounts of O_2_. A possible explanation for the low O_2_ evolution observed with the more potent photosensitizers is that they generate highly reactive Pd^IV^ oxide species that mainly take part in a degradation process of the photosensitizer instead of producing O_2_.

To rule out that the low O_2_ evolution observed with the stronger photosensitizers did not originate from a deactivation of the Pd nanocatalyst, a photochemical experiment with the milder [Ru(bpy)_3_]^2+^ photosensitizer was performed with a sample of the Pd nanocatalyst that had been recovered from a photochemical run with the strong photosensitizer [Ru(bpy)- (deeb)_2_]^2+^. The recovered Pd nanocatalyst gave rise to O_2_ evolution comparable to that of the unused Pd nanocatalyst (see Figure S10 in the Supporting Information), which confirmed that the strong photosensitizer [Ru(bpy)(deeb)_2_]^2+^ did not cause degradation or deactivation of the Pd nanocatalyst.

### Comparison with previous heterogeneous systems and mechanistic considerations

By directly comparing the performance of the developed Pd nanocatalyst in terms of activity and stability with that of previously reported heterogeneous WOCs under chemical[33,43,46] and photochemical conditions,[32,37,39,41,43,45,48] it is evident that the current catalytic system compares well with the current state-of-the-art heterogeneous WOCs. The performance of the Pd nanocatalyst in mediating H_2_O oxidation can be attributed to its beneficial nanostructure, which consists of small Pd nanoparticles and grants access to a reactivity that is not present in bulk Pd metal. To demonstrate this unique reactivity, chemical experiments with commercially available Pd/C and Pd(OH)_2_/C were performed with [Ru(bpy)_3_](PF_6_)_2_ as the oxidant. These conventional heterogeneous Pd catalysts were characterized beforehand with HAADF-STEM and were confirmed to possess irregular distributions of Pd atoms in broad size ranges (compare Figures S8 and S9 with images of the Pd nanocatalyst depicted in Figure S7; see the Supporting Information). For each reaction, the same molar amount of Pd was used, and these heterogeneous Pd catalysts only evolved negligible quantities of O_2_.

**Scheme 1:**
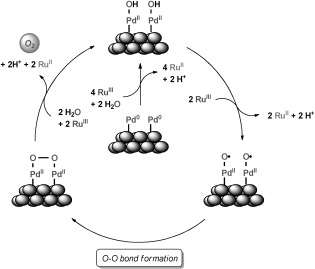
Proposed mechanism for the oxidation of H_2_O catalyzed by the Pd nanocatalyst.

To gain mechanistic insight into the Pd species involved in the H_2_O-oxidation catalysis, XPS was used to analyze the Pd nanocatalyst recovered from catalytic experiments with stoichiometric amounts of [Ru(bpy)_3_]^3+^ (see Figure S6a in the Supporting Information). By comparing the XPS spectrum of the unused Pd nanocatalyst (see Figure S6b in the Supporting Information) with that of the used catalyst, it is obvious that there is a substantial increase of the component that belongs to PdO/Pd(OH)_2_ (i.e., at 336.3 eV) in the latter spectrum. In comparison to the unused Pd nanocatalyst, for which the PdO/Pd(OH)_2_ signal only constituted approximately 20 % of the total Pd signal, the recovered catalyst measured up to approximately 80–90 % oxidized Pd. Taken together, the results obtained from the photocatalytic experiments with different photosensitizers and XPS suggest that the Pd nanocatalyst can potentially operate through the route depicted in Scheme 1. The mechanism starts with the oxidation of the Pd^0^ surface atoms into Pd^II^ centers (here illustrated as Pd^II^—OH). The surface-bound Pd^II^ species can then undergo another one-electron oxidation to generate a formal Pd^II^–oxyl radical species (equivalent to Pd^III^—O). Given the proximity of the Pd centers on the catalyst surface, it seems likely that at least two sites are cooperatively involved in the catalytic mechanism, in analogy with the mechanism of, for example, the so-called “blue dimer”.[49,64,65] Thus, two adjacent Pd centers can undergo O—O coupling to generate the O—O bond through a biradical coupling mechanism (Scheme 1). This step is in analogy to the operation of a few artificial homogeneous WOCs.[49,64,65] Liberation of O_2_ with subsequent oxidation results in the regeneration of the Pd^II^ hydroxide/oxide species.

However, the generated Pd^II^–oxyl radical species could also be susceptible to nucleophilic attack. Therefore, it is not possible to exclude the possibility that O_2_ bond formation event occurs through nucleophilic attack by H_2_O on this Pd^II^–oxyl radical species, which has been shown to occur for a variety of artificial WOCs.[49] From the generated Pd^II^–superoxo species, O_2_ can be evolved, which closes the catalytic cycle and retrieves the Pd^II^ species. A mechanistic investigation is currently underway in our laboratory to establish the exact mechanism of O_2_ evolution by this Pd nanocatalyst.

## Conclusion

We have disclosed an application of a Pd nanocatalyst in a chemically driven H_2_O oxidation with Ce^IV^ and [Ru(bpy)_3_]^3+^ as the terminal oxidants. The catalyst consists of Pd nanoparticles in the size range of 1.5–2.6 nm, immobilized on an amino-functionalized MCF, which confers a reactivity that has not been achieved previously with molecular Pd complexes or bulk Pd metal. Moreover, the TOF (2.7×10^−2^ s^−1^ per bulk Pd content) calculated for the Pd nanocatalyst from catalytic H_2_O oxidation experiments with the one-electron oxidant [Ru(bpy)_3_]^3+^ is comparable with the majority of reported state-of-the art metal oxide catalysts for H_2_O oxidation. Gratifyingly, we could also show that the Pd nanocatalyst is relevant for integration into photosynthetic devices by demonstrating that this catalyst can mediate photochemical H_2_O oxidation when [Ru(bpy)_3_]^2+^ is used as the photosensitizer. In addition to its high catalytic activity, this silica-based Pd nanocatalyst also displayed several of the typical features associated with heterogeneous catalysts, such as a simple and cost-effective synthesis, high chemical stability, good recyclability, and a low degree of metal leaching. The results obtained herein suggest that the use of nanostructured transition metal species as WOCs holds great promise because they offer novel strategies to access highly efficient catalytic materials that can be incorporated into photoinduced H_2_O-splitting systems for the green and sustainable production of solar fuels.

## Experimental Section

### Materials

[Ru(bpy)_3_](PF_6_)_3_ was prepared according to a previously reported procedure.[66] All the other reagents, including solvents, were obtained from commercial suppliers and used directly without further purification. All the solvents were dried by using standard methods when needed.

### Methods

FTIR spectra were recorded on a Perkin-Elmer Spectrum One spectrometer, with samples prepared as KBr discs. The physical properties of the mesoporous materials were determined from N_2_ adsorption/desorption isotherms on an ASAP 2010 instrument. The Pd nanocatalyst was analyzed for Pd leaching by using inductively coupled plasma (Medac Ltd, Analytical and Chemical Consultancy Services, UK) and the size/distribution of the palladium nanoparticles was assessed by scanning transmission electron microscopy (STEM). For TEM analyses, small amounts of ground Pd nanocatalyst were added to EtOH, ultrasonicated, and a few drops of the resulting slurry were deposited onto a copper TEM grid with amorphous-carbon supporting films (SPI Supplies Inc.). Samples were dried thoroughly before insertion into the microscope column. For the particle analysis, high-angle annular dark-field STEM (HAADF-STEM) was performed on a 200 kV electron microscope with a Schottky field-emission gun (JEOL JEM-2100). The HAADF-STEM images were recorded using a JEOL ADF detector. The camera length was 8 cm and the incident beam probe size was approximately 0.2 nm. The gain of the detector was kept constant throughout the experiments on all the samples. Because the contrast of Pd in the HAADF-STEM images is apparently stronger than that of the MCF support, particle-size measurements were possible by careful adjustment of the background threshold. Imaging and particles-size analysis were carried out by Gatan Digital Micrograph (Gatan Inc.). X-ray photoelectron spectroscopy (XPS) was used to determine the structure and oxidation states of the Pd nanoparticles on the AmP-MCF material, and these analyses were recorded by using a Kratos AXIS UltraDLD x-ray photoelectron spectrometer. The samples were analyzed with a monochromatic aluminum X-ray source. The analysis area was approximately 1 mm^2^ (most of the signal is from an area of 700×300 μm).
