# Supplementary material for: Well-Defined Palladium Nanoparticles Supported on Siliceous Mesocellular Foam as Heterogeneous Catalysts for the Oxidation of Water
Source: Chemistry. 2015 Mar 16;21(15):5909–15. doi: 10.1002/chem.201406279 (PMC4464534; doi:10.1002/chem.201406279)
Supplement: Supplementary file 1 [file chem0021-5909-sd1.pdf]

CHEMISTRY
A European Journal

Supporting Information

Well-Defined Palladium Nanoparticles Supported on Siliceous Mesocellular Foam as Heterogeneous Catalysts for the Oxidation of Water

Oscar Verho,[a] Torbjörn Åkermark,[a] Eric V. Johnston,[a] Karl P. J. Gustafson,[a] Cheuk-W. Tai,[b, c] Henrik Svengren,[b] Markus D. Kärkäs,*[a] Jan-E. Bäckvall,*[a] and Björn Åkermark*[a]

chem_201406279_sm_miscellaneous_information.pdf

Preparation of the mesocellular foam (MCF). The MCF material was synthesized by a modified literature procedure.[S1] Triblock copolymer PEG-PPG-PEG Pluronic® P-123 (4.0 g) was dissolved in an aqueous HCl solution (10 mL conc. HCl in 65 mL H_2O). 1,3,5-Trimethylbenzene (3.4 mL) was subsequently added to this mixture and the resulting emulsion was vigorously stirred at 37-40 °C for 2 h. Tetraethoxysilane (9.2 mL) was added to the reaction and it was allowed to stir for additional 5 min. The resulting solution was then aged for 20 h at 40 °C under static conditions. NH_4F (46 mg) dissolved in H_2O (5.0 mL) was subsequently added and the reaction was stirred for 10 min, before it was sealed, heated to 100 °C, and allowed to age further for 24 h. The solution was allowed to reach room temperature and was thereafter filtered. The resulting MCF composite precipitate was extensively washed with H_2O (2.0 L) and EtOH (1.0 L). The resulting powder was dried under vacuum for 24 h before it was calcined at 560 °C (temp. gradient 300 °C/h) in air for 6 h to remove the remaining organic precursors.

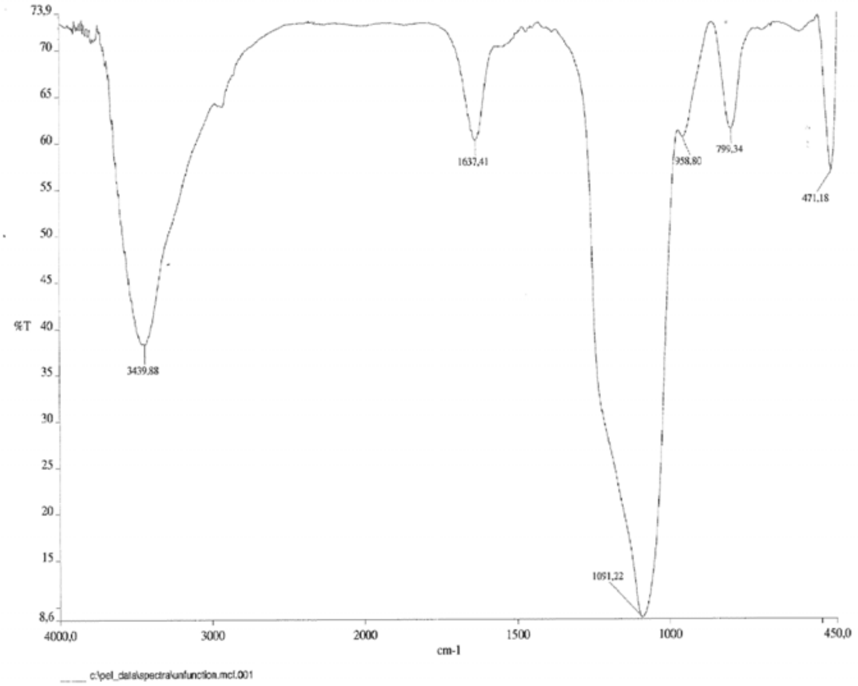

Figure S1. FTIR spectra of the synthesized MCF. FTIR (cm^{-1}): 3440 (O-H stretch), 1637 (C-O bend from carbon impurities in the MCF structure), 1091 (Si-O-Si stretch), 799 (Si-O bend), 471 (Si-O out of plane).

Aminopropyl-grafting of the MCF. The grafting of the MCF material with aminopropyl groups was done in accordance with a previously reported procedure.[S2] The unfunctionalized MCF material (2.0 g) was placed in a 100 mL round-bottom flask and dried under vacuum for 4 h. Dry toluene (40 mL) was then added and the resulting mixture was stirred under Ar atmosphere until a homogeneous suspension was obtained. (3-aminopropyl)trimethoxysilane (5.6 mL) in dry toluene (20 mL) was added to the mixture and the reaction was first stirred at room temperature for 5 min and then at 108 °C for 48 h. Afterwards, the reaction was allowed to reach room temperature, filtered and washed with toluene (400 mL), EtOH (250 mL) and CH_2Cl_2 (500 mL). The solid material was resuspended in EtOH (40 mL) and stirred at 60 °C for 16 h, to remove unreacted silane species from the internal pore system. The resulting solution was filtered, washed with EtOH (250 mL) and CH_2Cl_2 (250 mL), and dried over night under vacuum to give the functionalized AmP-MCF material.

By employing the Barrett-Joyner-Halenda method for isotherm analysis, the average pore size and window size of the pristine AmP-MCF material were determined to 26.0 nm and 13.6 nm, respectively.[S2] The specific pore volume and surface area for the pristine AmP-MCF were calculated, using Brunauer-Emmett-Teller (BET) surface area analysis, to 1.65 cm^3 g^{-1} and 380 m^2 g^{-1}, respectively.[S2] The amine loading in the resulting AmP-MCF material was determined from elemental analysis of the nitrogen content and was measured to 1.9 wt%.

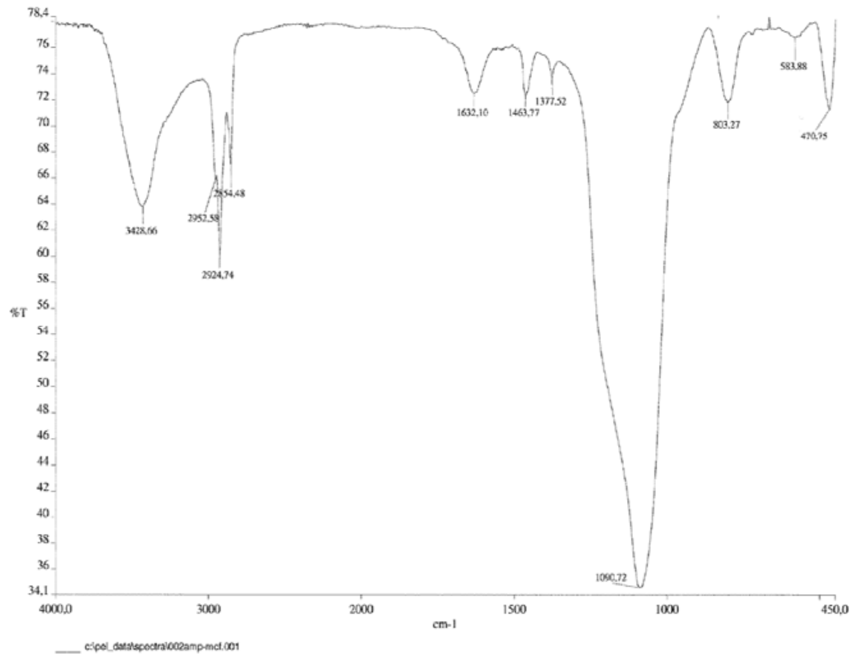

Figure S2. FTIR spectra of the AmP-functionalized MCF. FTIR (cm^{-1}): 3429 (O-H stretch), 2953 (C_{sp3}-H stretch), 2925 (C_{sp3}-H stretch), 2854 (C_{sp3}-H stretch), 1637 (C-O bend from residual carbon impurities in the MCF structure), 1464 (C_{sp3}-H bend), 1378 (C-N stretch), 1091 (Si-O-Si stretch), 803 (Si-O bend), 471 (Si-O out of plane).

Preparation of the Pd nanocatalyst. The Pd nanocatalyst was prepared by a modified literature procedure.[S2] Amino-functionalized MCF (0.50 g) was suspended in deionized H_2O (15 mL, pH 9.0, pH adjusted by 0.1 M LiOH) in a falcon tube and stirred for 10 min. Dry Li_2PdCl_4 (214.9 mg, 0.82 mmol) in deionized H_2O (10 mL, pH 9) was added to the suspension of amino-functionalized MCF. The solution was stirred for 24 h at room temperature, after which the Pd^{II}-AmP-MCF precatalyst was separated by centrifugation (4000 rpm for 9 min). To remove non-complexated Pd, the solid material was washed with H_2O (3 × 50 mL), using centrifugation technique. The Pd^{II}-AmP-MCF was then resuspended in deionized H_2O (15 mL) and divided into two falcon tubes. $NaBH_4$ (155 mg, 8.20 mmol) in H_2O (3 mL) was added dropwise to each falcon tube and the reactions were stirred at room temperature for 30 min. The tubes were then filled up with deionized H_2O, centrifuged (4100 rpm for 9 min), and the resulting supernatant was poured off. This was repeated three times with H_2O and three times with acetone to give the Pd nanocatalyst, which was dried under vacuum before use.

By employing the Barrett-Joyner-Halenda method for isotherm analysis, the average pore size and window size of the Pd nanocatalyst were determined to 18.4 nm and 12.0 nm, respectively. The specific pore volume and surface area for the Pd nanocatalyst were calculated, using BET surface area analysis, to 1.55 cm^3 g^{-1} and 341 m^2 g^{-1}, respectively. The Pd and N loading in the resulting Pd nanocatalyst was measured from elemental analysis to 7.9 wt% and 1.4 wt%, respectively.

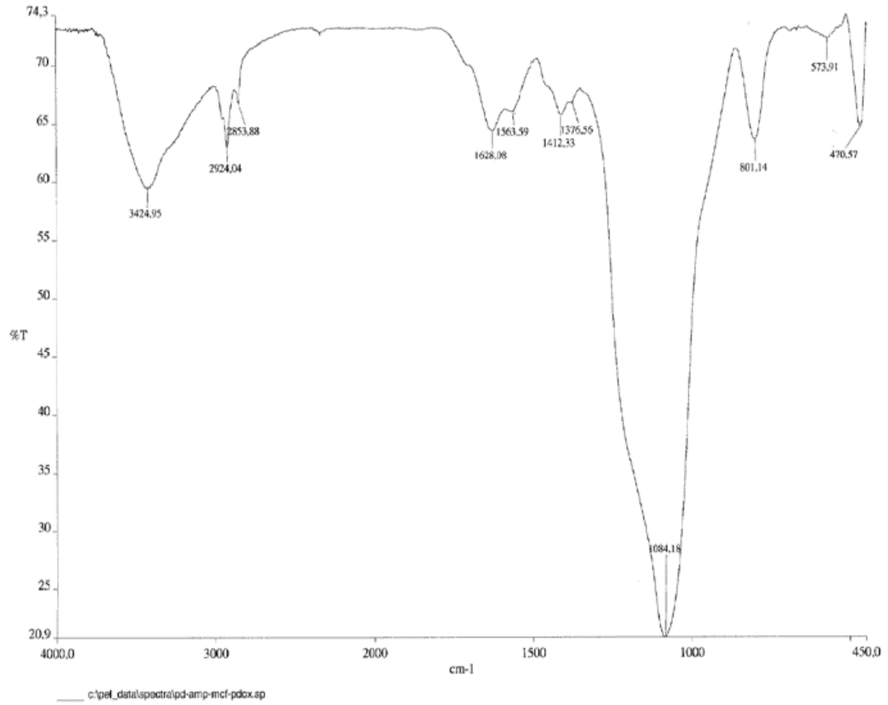

Figure S3. FTIR spectra of the synthesized Pd nanocatalyst. FTIR (cm^{-1}): 3425 (O-H stretch), 2924 (C_{sp3}-H stretch), 2854 (C_{sp3}-H stretch), 1628 (C-O bend from residual carbon impurities in the MCF structure), 1564 (N-H bend), 1412 (C_{sp3}-H bend), 1377 (C-N stretch), 1084 (Si-O-Si stretch), 801 (Si-O bend), 471 (Si-O out of plane).

Gas analysis by mass spectrometry (MS). Oxygen evolution was measured by MS, where the mass spectrometer consisted of three separate parts connected by gas valves; a reaction chamber, a gas handling system (GHS), and a mass spectrometer (MKS Spectra Products, Microvision Plus, 0-100 mass units) in ultra high vacuum (base pressure $2 \cdot 10^{-10}$ mbar). A rough pump is used to evacuate the GHS, so the pressure can be regulated within 0.1-1000 mbar. The enclosed volume in the reaction

chamber is continuously probed by the mass spectrometer by an inlet through the leak valve. A ca 1 cm thick rubber gasket has been added to the system and permits injection of solutions containing reactants into the reaction chamber, essentially without any leakage of the external atmosphere. Any air leakage is continuously followed by the mass spectrometer (by increase of both O_2 and N_2). In order to avoid splashing when injecting the solution a pressure of ~40 mbar is needed and in this study we have filled the enclosed volume with He to obtain a pressure of ~40 mbar.)

The change over time of the measured pressure of masses 0-100 in the mass spectrometer is thus converted to the amount in the enclosed volume in two steps. The first step is to convert the pressure in the mass spectrometer to the pressure in the enclosed volume. This conversion is done by calibration of the system, i.e. measuring the response in the MS to different pressures in the enclosed volume. In the second step the pressures in the enclosed volume are converted to the amounts of the different gases by using the gas laws, which makes it possible to determine the production of gases with masses 1-100 quantitatively.

Procedure for O_2 evolution measurements. *(a) Chemical oxidation with Ce^{IV}*: In a typical run the Pd nanocatalyst (0.60 mg Pd nanocatalyst, 7.9 wt% Pd, 0.45 μmol Pd) and $[(NH_4)_2Ce(NO_3)_6]$ (61 mg, 0.11 mmol) were placed in the reaction chamber and the reaction chamber was evacuated with a rough pump. ~40 mbar He was then introduced into the system. After an additional 5 min, deoxygenated H_2O (1.0 mL, MilliQ), bubbled with N_2 for at least 5 min, was injected into the reaction chamber. The generated oxygen gas was then measured and recorded versus time by MS. The results from this experiment are shown in Figure 3.

(b) Chemical oxidation with $[Ru(bpy)_3]^{3+}$: In a typical run $[Ru(bpy)_3](PF_6)_3$ (45 mg, 45 μmol) and the Pd nanocatalyst (0.25 mg Pd nanocatalyst, 7.9 wt% Pd, 0.19 μmol Pd) were placed in the reaction chamber and the reaction chamber was evacuated with a rough pump. ~40 mbar He was then introduced into the system. After an additional 5 min, deoxygenated aqueous phosphate buffer solution (0.1 M, pH 7.2, 0.50 mL), bubbled with N_2 for at least 5 min, was injected into the reaction chamber. The generated oxygen gas was then measured and recorded versus time by MS. The results from these experiments are shown in Figure 4a.

(c)Photochemical oxidation using $[Ru(bpy)_3]^{2+}$: In a typical run $[Ru(bpy)_3]^{2+}$-type photosensitizer (400 µM, 0.20 µmol), sodium persulfate (24 mM, 12 µmol) and Pd nanocatalyst (0.50 mg, 7.9 wt% Pd, 0.37 µmol Pd) were placed in the reaction chamber and the reaction chamber was evacuated with a rough pump and all MeCN was evaporated. ~40 mbar He was then introduced into the system. After an additional 5 min, deoxygenated aqueous phosphate buffer solution (0.1 M, pH 7.2, 0.50 mL), bubbled with N_2 for at least 5 min, was injected into the reaction chamber and the reaction was irradiated by visible light. To avoid heating of the reaction by the light source, the reaction vessel was placed in a water bath and cooled with a small flow of water. The generated oxygen gas was measured and recorded versus time by MS. The results from these experiments are shown in Figure 5 and Figure S4.

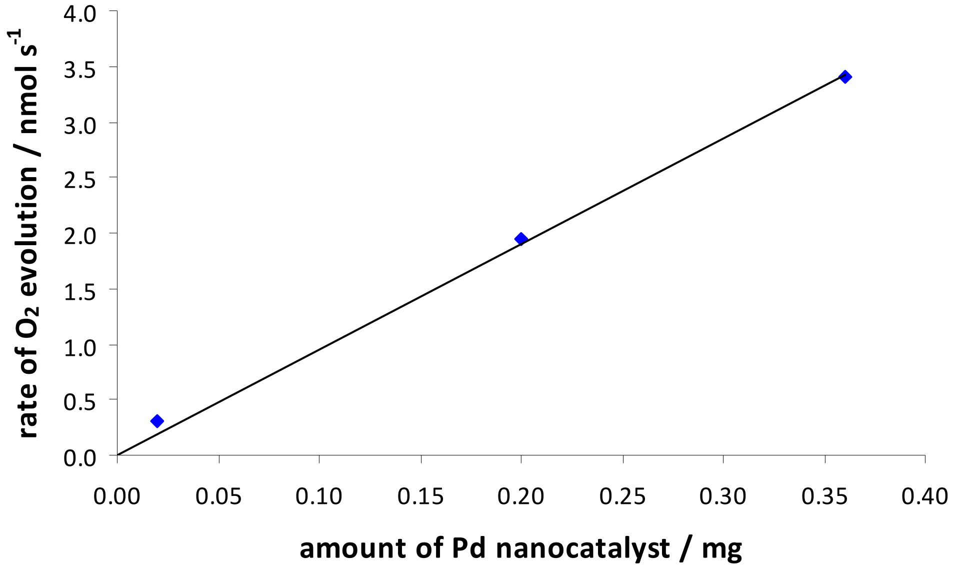

Figure S4. Kinetic curve for chemical O_2 evolution using different amount of Pd nanocatalyst. Conditions: Experiments were performed in an aqueous phosphate buffer solution (0.1 M, pH 7.2, 0.50 mL) containing Pd nanocatalyst (0.02, 0.20, 0.36 mg) and $[Ru(bpy)_3](PF_6)_3$ (7.2 mg, 7.2 µmol).

^{18}O-Isotopic experiments. $[Ru(bpy)_3](PF_6)_3$ (5.3 mg, 5.3 µmol) and the Pd nanocatalyst (0.37 mg, 7.9 wt% Pd, 0.28 µmol Pd) were placed in the reaction chamber and the reaction chamber was evacuated with a rough pump. ~40 mbar He was then introduced into the system. After an additional 2 min, deoxygenated aqueous phosphate buffer solution (0.1 M, pH 7.2, 0.50 mL, 16% $H_2{}^{18}O$) was injected

into the reaction chamber. The generated oxygen gas was then measured and recorded versus time by MS. The results from this experiment are shown in Figure S5.

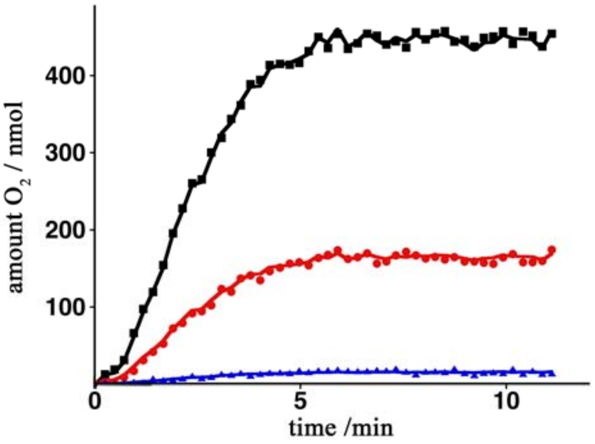

Figure S5. Chemical H_2O oxidation catalyzed by the Pd nanocatalyst in isotopically labeled H_2O ($H_2{}^{18}O$). Experimental conditions: Reactions were carried out in an aqueous phosphate buffer solution (0.1 M, pH 7.2, 0.50 mL, 16% $H_2{}^{18}O$) in the presence of Pd nanocatalyst (0.37 mg Pd nanocatalyst, 7.9 wt% Pd, 0.28 μmol Pd) and $[Ru(bpy)_3](PF_6)_3$ (5.3 mg, 5.3 μmol). (■) $^{16,16}O_2$, (●) $^{16,18}O_2$, (▲) $^{18,18}O_2$. The solid lines are statistical distributions of $^{16,16}O_2$, $^{16,18}O_2$ and $^{16,18}O_2$ calculated from the isotopic concentration of H_2O (16% ^{18}O) and the total amount of produced O_2.

XPS-analysis of Pd nanocatalyst recovered from a catalytic experiment using $[Ru(bpy)_3](PF_6)_3$. A chemical H_2O oxidation experiment was conducted with Pd nanocatalyst (11 mg, 7.9 wt% Pd, 8.0 μmol Pd) and $[Ru(bpy)_3](PF_6)_3$ (125 mg, 125μmol) in deoxygenated phosphate buffer solution (0.1 M, pH 7.2, 0.50 mL) for 10 min). After the catalytic experiments, the reaction solution was transferred to a Falcon tube (15 mL), suspended in MeCN (5 mL) and centrifuged (4000 rpm, 10 min). The supernatant was removed and the Pd nanocatalyst was washed with additional MeCN (2 x 5 mL) and acetone (3 x 5 mL) using centrifugation technique. The Pd nanocatalyst was dried *in vacuo* overnight, before it was subjected to XPS-analysis. By comparing the recorded XPS spectrum of the recovered Pd nanocatalyst (Figure S6a) to that of an unused sample (Figure S6b), a clear increase of the peak belonging to Pd^{II} oxide (PdO) at 336.3 eV together with a decrease of the peak belonging to Pd^0 at

335.1-335.5 eV were observed.[S3] In unused Pd nanocatalyst the PdO signal was found to only constitute ~20% of the total Pd signal, while it in the recovered catalyst measured ~80-90% of the total Pd signal.

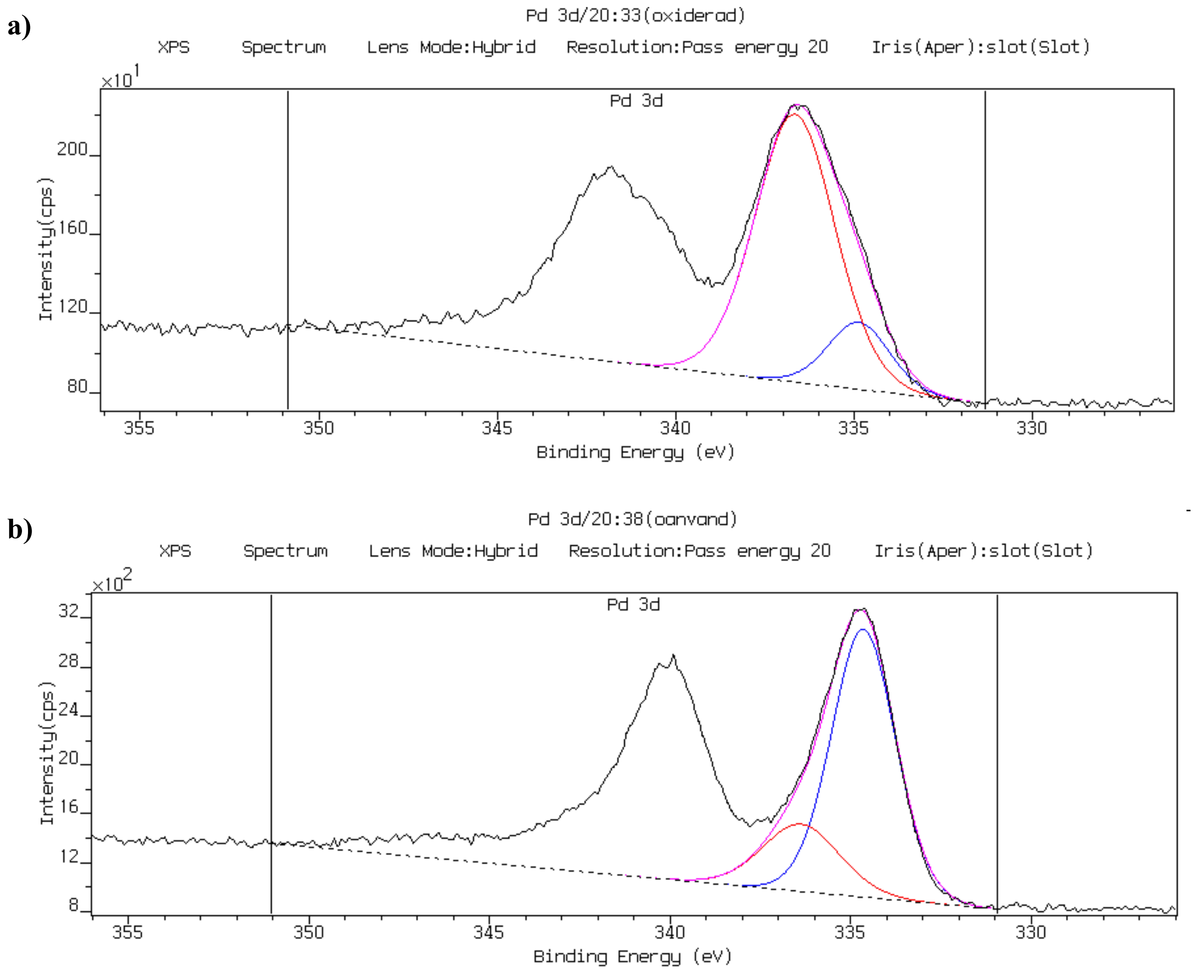

Figure S6. XPS spectra of recovered and unused Pd nanocatalyst. (—) combined Pd peak, (—) Pd^{II} component, (—) Pd^0 component. (a) Recovered catalyst from a chemical H_2O oxidation experiment using $[Ru(bpy)_3]^{3+}$ as the chemical oxidant. (b) Unused Pd nanocatalyst.

Procedure for recycling of the Pd nanocatalyst in catalytic H_2O oxidation experiments. After the catalytic experiments, the reaction solution was transferred to a Falcon tube (15 mL), suspended in H_2O/MeCN (1:1, 5 mL) and centrifuged (4000 rpm, 10 min). The supernatant was removed and the Pd nanocatalyst was re-suspended in H_2O/MeCN (1:1, 1.5 mL), transferred to an Eppendorf tube (2 mL)

and centrifuged (10 000 rpm, 3 min). This was repeated 3 times, followed by washing with acetone (3 x 2 mL) using centrifugation technique. The resulting solid was dried under vacuum, before it was used again in a new catalytic experiment. The results from these experiments are shown in Figure 4b.

HAADF-STEM characterization of the Pd nanocatalyst

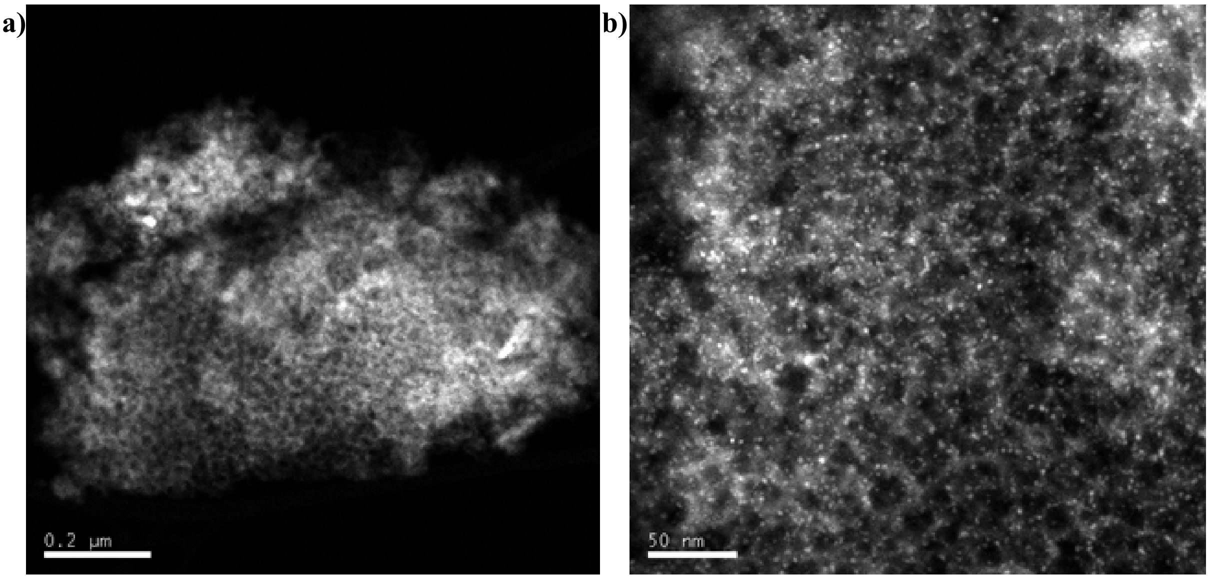

Figure S7. HAADF-STEM images of the Pd nanocatalyst. (a) with 0.2 µm scale bar. (b) with 50 nm scale bar.

HAADF-STEM characterization of commercial heterogeneous Pd catalysts

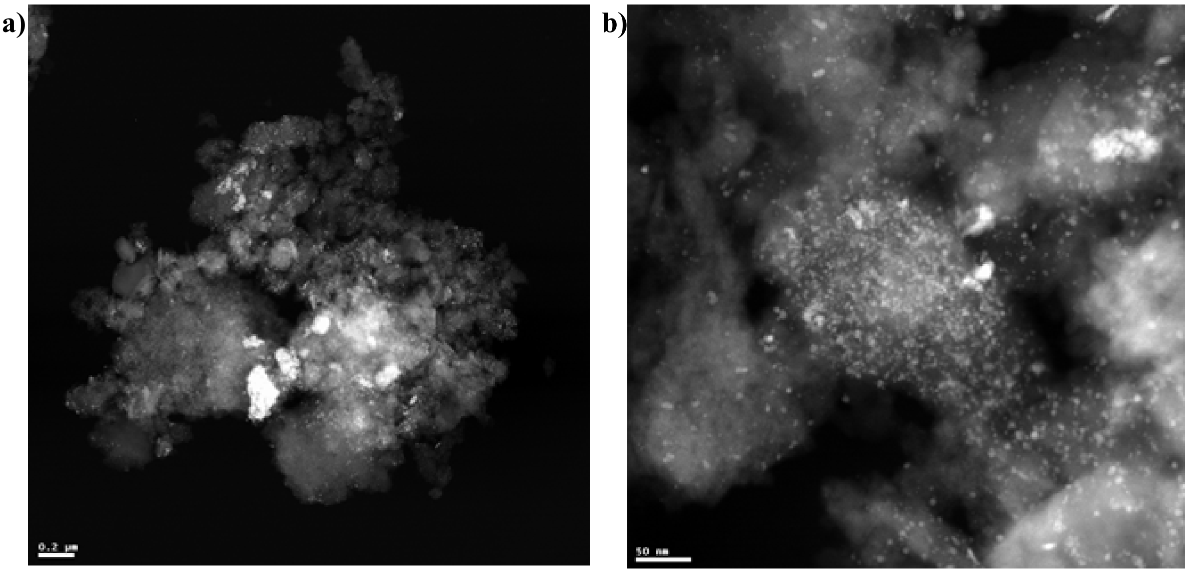

Figure S8. HAADF-STEM images of commercial Pd@C (10 wt%). (a) with 0.2 µm scale bar. (b) with 50 nm scale bar.

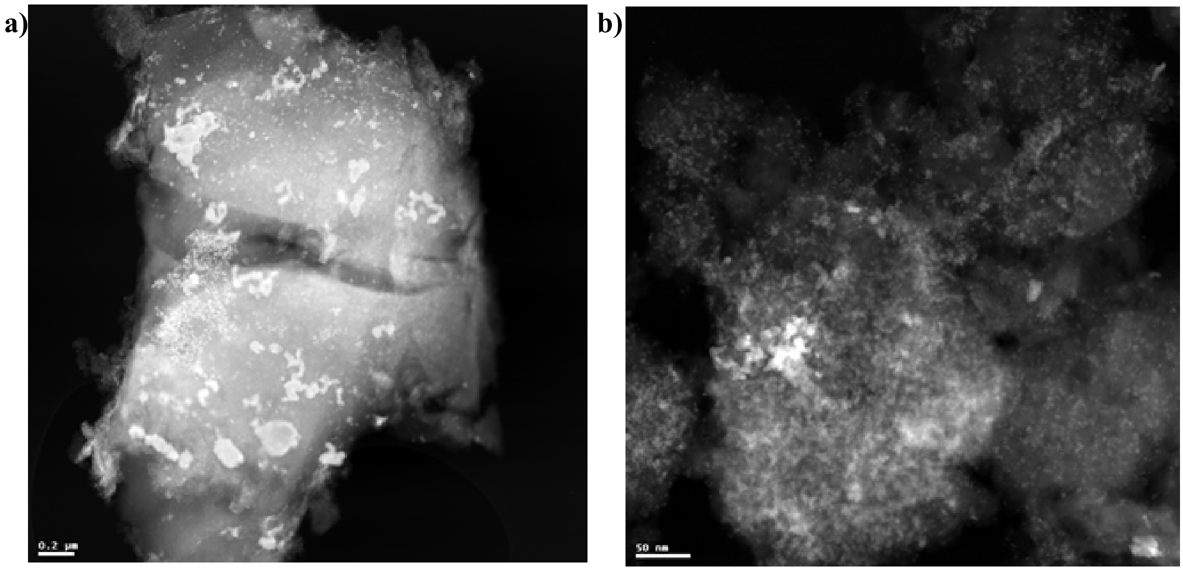

Figure S9. HAADF-STEM images of commercial $Pd(OH)_2$@C (20 wt%). (a) with 0.2 µm scale bar. (b) with 50 nm scale bar.

Consecutive photochemical oxidation using [Ru(bpy)(deeb)$_2$]$^{2+}$ followed by [Ru(bpy)$_3$]$^{2+}$. Pd nanocatalyst (1.1 mg, 7.9 wt% Pd, 0.82 μmol Pd), [Ru(bpy)(deeb)$_2$](PF$_6$)$_2$ (0.40 μmol) and sodium persulfate (15 mg, 64 μmol) were placed in the reaction chamber and the reaction chamber was evacuated with a rough pump. ~40 mbar He was then introduced into the system. After an additional 2 min, deoxygenated aqueous phosphate buffer solution (0.1 M, pH 7.2, 0.5 mL), bubbled with N_2 for at least 15 min, was injected into the reaction chamber. The generated oxygen gas was then measured and recorded versus time by MS. After the catalytic experiments, the reaction solution was transferred to a Falcon tube (15 mL), suspended in MeCN (5 mL) and centrifuged (4000 rpm, 10 min). The supernatant was removed and the Pd nanocatalyst was washed with additional MeCN (2 x 5 mL) and acetone (3 x 5 mL) using centrifugation technique. The Pd nanocatalyst was dried *in vacuo* overnight, before it was reused in a subsequent reaction cycle, using [Ru(bpy)$_3$](PF$_6$)$_3$ (0.20 μmol) and sodium persulfate (5.2 mg, 22 μmol). The generated O_2 was once again measured and recorded versus time by MS. The results from these experiments are shown in Figure S10.

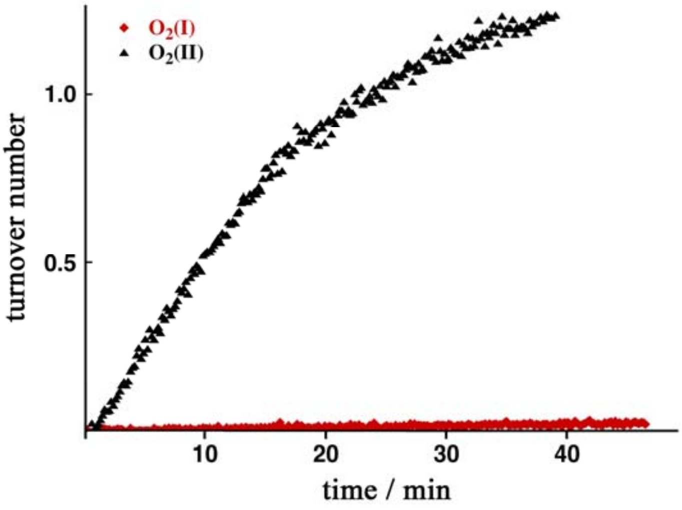

Figure S10. Kinetic curves for photochemical O_2 evolution by the Pd nanocatalyst versus time. (I) Using fresh Pd nanocatalyst and [Ru(bpy)(deeb)$_2$](PF$_6$)$_2$ as photosensitizer. (II) Using recycled Pd nanocatalyst and [Ru(bpy)$_3$](PF$_6$)$_2$ as photosensitizer.

Table S1. Calculated yields of O_2 based on oxidant.

**Amount of Pd nanocatalyst (µmol)**	**Amount of oxidant (µmol)**	**Evolved O_2 (µmol)**	**Yield of O_2 based on oxidant (%)[†]**
0.45	110 (Ce^{IV})	9.0	33
0.19	45 ($[Ru(bpy)_3]^{3+}$)	0.57	5.1
0.015	7.0	0.15	8.6
0.27	5.3	0.70	53

[†] Yield of O_2 based on oxidant = 4 · amount of O_2 / amount of oxidant.

References

S1. Y. Han, S. S. Lee, J. Y. Ying, *Chem. Mater.* **2006**, *18*, 643-649.

S2. E. V. Johnston, O. Verho, M. D. Kärkäs, M. Shakeri, C.-W. Tai, P. Palmgren, K. Eriksson, S. Oscarsson, J. E. Bäckvall, *Chem. Eur. J.* **2012**, *18*, 12202-12206.

S3. L. Chen, A. Yelon, E. Sacher, *J. Phys. Chem. C* **2011**, *115*, 7896-7905.
